# Predictors of post-stroke body temperature elevation

**DOI:** 10.1186/s12883-017-1002-3

**Published:** 2017-12-13

**Authors:** Rebecca Ruborg, Karin Gunnarsson, Jakob O. Ström

**Affiliations:** 1 Department of Neurology, Faculty of Medicine and Health, Örebro University, Örebro, Sweden; 2 Department of Clinical Chemistry and Department of Clinical and Experimental Medicine, Linköping University, Linköping, Sweden; 3 Region Örebro Län, Neuro- och rehabmedicinska kliniken, Södra Grev Rosengatan, 701 85 Örebro, Sweden

**Keywords:** Stroke, Fever, Body temperature, Infection

## Abstract

**Background:**

Growing evidence indicates that elevated body temperature after stroke is associated with unfavorable outcome. The aim of the current study was to investigate which factors predict temperature elevation within 48 h of stroke onset. Specifically, we hypothesized that temperature elevation would be associated with stroke symptom severity and that hemorrhagic stroke would cause a more pronounced temperature increase compared to ischemic stroke.

**Methods:**

The medical records of 400 stroke patients were retrospectively reviewed. Multiple linear regression analysis was used to determine which factors were associated with elevated body temperature.

**Results:**

Several factors were significantly associated with peak body temperature (the highest recorded body temperature) within 48 h of stroke onset: stroke severity measured by the National Institutes of Health Stroke Scale (NIHSS) (regression coefficient; (RC) 0.022), female gender (RC 0.157), tympanic/non-rectal temperature reading (RC −0.265), swallowing difficulties (RC 0.335), intubation (RC 0.470), antipyretic treatment (RC 0.563), and C-reactive protein > 50 or signs of infection at admission (RC 0.298). Contrary to our expectations, patients with intracerebral hemorrhage did not have higher peak body temperatures than patients with ischemic stroke.

**Conclusions:**

In conclusion, temperature elevation within the first 48 h of stroke onset is common, can be partially predicted using information at admission and is strongly associated with stroke severity. The strong association with stroke severity may, at least partly, explain the previously described association between post-stroke temperature elevation and unfavorable outcome.

## Background

Different factors have been shown to be associated with recovery from stroke, for example body temperature after the incident [1–3]. In addition to being at risk of bacterial infections such as pneumonia, stroke patients often suffer from temperature elevations without an identifiable infection; possibly endogenous fever due to direct effects of the infarction or hemorrhage on the brain [4–6]. Although numerous studies regarding body temperature after stroke have been published, treatment guidelines for post-stroke temperature elevation are ambiguous and no firm clinical consensus has been reached [7].

A profound understanding of the pathophysiology of body temperature elevation after stroke onset is pivotal for the development of successful treatment strategies. Clues regarding endogenous temperature elevation are especially important, since distinguishing between endogenous and infectious fever is a common clinical dilemma. One important piece of this puzzle could be provided by mapping factors that predispose to temperature elevation. For example, if it was known that patients with intracerebral hemorrhage are more prone to early temperature elevation compared to patients with ischemic stroke, then this could indicate that the elevation in temperature is partly mediated by the intrathecal release of blood products and that higher non-infectious temperature elevations are to be expected in these patients. Previously, stroke severity, as well as various patient characteristics such as age and gender, has been shown to be associated with post-stroke temperature elevation [1, 8]. However, several important questions, such as whether hemorrhagic or ischemic stroke causes the most temperature elevation, remain unanswered.

Therefore, the aim of this study was to investigate which factors were associated with peak body temperature (defined as the highest recorded body temperature for each patient) within 48 h of stroke onset. Specifically, we hypothesized that peak body temperature would be associated with stroke severity and that hemorrhagic stroke would cause a more pronounced temperature increase compared to ischemic stroke.

## Methods

**Fig. 1:**
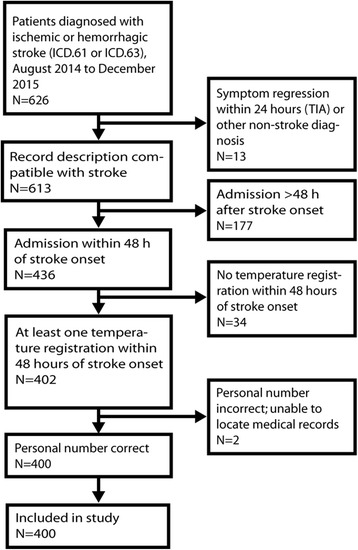
Flow chart showing the inclusion process. ICD = International Classification of Diseases and Related Health Problems; TIA = transient ischemic attack

This study was a retrospective review of the medical records at the University Hospital in Örebro. A medical secretary collected the names of patients diagnosed with International Classification of Diseases (ICD) I61 and I63 from the Swedish Stroke Register during 2014 and 2015. Inclusion criteria were cerebral infarction or intracerebral hemorrhage together with at least one temperature reading within the first 48 h of clinical stroke onset (median number of temperature registrations per patient and day was 3). Altogether, 400 patients with ischemic or hemorrhagic stroke were included from August 2014 to December 31st, 2015. Patients with transient symptoms within 24 h were assessed as transient ischemic attack (TIA) and were excluded, even if there were signs of ischemia on computed tomography or if treatment with thrombolysis had been given. A flow chart of the inclusion process is presented in Fig. 1.

Data including patient characteristics, stroke subtype, laboratory parameters, previous and newly initiated treatments and temperature readings was extracted from the medical records in accordance with a prewritten protocol (all temperature readings during 48 h were noted and the highest temperature readings within time interval were used in the analyses). If smoking was explicitly mentioned in the medical record, the patient was classified as a smoker; if no smoking was reported or smoking was not mentioned, it was assumed that the patient did not smoke. If the exact time of the stroke onset was uncertain, the time-point at which the patient had last been seen free from symptoms was considered as stroke onset. In cases where exact timing of stroke onset was lacking, time-points were stipulated according to a prespecified algorithm: symptom onset described as in the “morning”, “afternoon” and “evening” was interpreted as stroke onset at 8 am, 4 pm, and 8 pm, respectively. For patients who had woken up with stroke symptoms with no recorded time of going to bed, stroke onset was assumed to have been at 10 pm. If the patients had had a National Institutes of Health Stroke Scale (NIHSS) score documented at arrival, the score was noted, whereas in patients without documented NIHSS score, neurological status was converted to NIHSS score manually in accordance with a protocol. Where a patient had suffered from stroke earlier and had residual symptoms that were described in the medical records, this was subtracted from the current NIHSS score to get an estimation of the severity of the current stroke. Where hypertensive medication or statins had been prescribed during hospitalization, these were regarded as signs of hypertension or hyperlipidemia. At the intensive care unit, temperature had been measured using a urinary catheter, which is comparable to rectal temperature measurements, and regarded as such in the statistical analysis. If a new stroke had occurred during hospital stay, the second event was excluded from the study. C-reactive protein (CRP) more than 50 was counted as a sign of infection.

### Statistics

Normally distributed data was presented with mean values and standard deviation (SD). Median, minimum and maximum values and/or interquartile range (IQR) were used for data not normally distributed. Frequencies were calculated for all categorical variables. Peak body temperature was defined for each patient as the highest recorded temperature within 48 h from stroke onset. Multiple linear regression analyses were performed using SPSS 23 (IBM Statistics 23 Armonk, NY, USA), assessing the effect of patient characteristics and stroke characteristics (including hemorrhagic/ischemic and NIHSS) on the outcome variable peak body temperature (treated as a continuous variable). Backward multiple regression analysis was performed using a *p*-value >0.2 to exclude non-significant variables from the regression model. A p-value <0.05 was considered statistically significant in the final regression analysis.

### Ethics

This study was a retrospective review of medical records at the University Hospital in Örebro and was performed with approval from the regional ethical committee in Uppsala, Sweden (2015/516). Written consent from the patients or their relatives to participate in the study was not obtained. However, when being registered with the Swedish Stroke Register, patients are informed of the possibility that the data could be used in studies, and of their right to withdraw their data from the register. The same 400 patients included here were also included in a study on predicting factors for mortality after stroke, simultaneously submitted for publication.

## Results

In total, 626 patients with cerebral infarction or intracerebral hemorrhage were reported by Örebro University Hospital to the Swedish Stroke Register between August 2014 and December 2015. Of these, 400 fulfilled the inclusion criteria and 226 were excluded. Patients with symptom regression within 24 h or a diagnosis that was not ischemic or hemorrhagic stroke were excluded as well as patients who arrived to the hospital more than 48 h after stroke onset, patients with no temperature reading within 48 h of stroke onset and patients whose medical records could not be found (Fig. 1). Baseline characteristics are summarized in Table 1.

**Table 1:** Baseline characteristics

Age, yrs. mean ± SD	77 ± 12.5
Women, *N* (%)	205 (51)
Smoking, *N* (%)	47 (12)
Anticoagulants at admission, *N* (%)	53 (13)
Antithrombotic therapy at admission, *N* (%)	145 (36)
Antipyretic drugs daily at admission, *N* (%)	91 (23)
Antipyretic drugs, when needed, at admission, *N* (%)	30 (8)
Antibiotics at admission, *N* (%)	17 (4)
Hypertension, *N* (%)	298 (75)
Diabetes, *N* (%)	90 (23)
Atrial fibrillation, *N* (%)	129 (32)
Hypercholesterolemia, *N* (%)	210 (53)
Previous stroke/TIA, *N* (%)	132 (33)
Malignancy, *N* (%)	40 (10)
Ischemic coronary heart disease/heart failure, *N* (%)	101 (25)
Chronic obstructive pulmonary disease/ other chronic lung disease, *N* (%)	41 (10)
Chronic inflammatory disease, *N* (%)	41 (10)
Chronic infection, *N* (%)	8 (2)

*SD* standard deviation, *TIA* transient ischemic attack

The mean of all patients’ peak body temperature readings within 48 h was 37.5 °C, the highest peak body temperature was 41.5 °C and the lowest peak body temperature was 34.9 °C. Temperature subgroups, signs of infection and other hospitalization parameters are summarized in Table 2. The median frequency/number of temperature readings per patient and day within 48 h of stroke onset was three.

**Table 2:** Parameters during hospitalization

NIHSS score at admission, *N* (%)	
0; *N* (%)	54 (14)
1–4; *N* (%)	185 (46)
5–15; *N* (%)	128 (32)
16–20; *N* (%)	9 (2)
> 21; *N* (%)	24 (6)
NIHSS score at admission, median (IQR)	3 (7)
Antibiotics at admission, *N* (%)	17 (4)
Temperature at admission, °C, mean ± SD	36.5 ± 3.8
CRP level at admission, mg/L, median (min- max)	3.5 (1–194)
Signs of infection or CRP > 50 at admission, *N* (%)	65 (16)
Stroke type, *N* (%)	
Infarction	351 (88)
Hemorrhage	49 (12)
Number of temperature readings median (min-max)	3 (1–27)
Peak body temperature within 48 h, *N* (%)	
< 35.5 °C	1 (0.3)
35.6–36.5 °C	24 (6)
36.6–37.5 °C	198 (50)
37.6–38.5 °C	140 (35)
38.6–39.5 °C	32 (8)
39.6–40.5 °C	5 (1)
40.6–41.5 °C	1 (0.3)
Temperature measurement method, *N* (%)	
Rectal	263 (66)
Tympanic	137 (34)
Thrombolysis, *N* (%)	23 (6)
Thrombectomy, *N* (%)	2 (0.5)
Decompression surgery, *N* (%)	2 (0.5)
Antipyretic treatment within 48 h of stroke onset, *N* (%)	201 (50)
Antibiotic treatment during hospital stay, *N* (%)	63 (16)
CRP > 50 during hospitalization, *N* (%)	34 (9)
Indwelling urinary catheter during hospitalization, *N* (%)	82 (21)
Positive swallowing test, *N* (%)	43 (11)
Undergoing intubation, *N* (%)	12 (3)
Signs of urinary tract infection- /positive urine cultures, *N* (%)	31 (8)
Signs of pneumonia or positive lung radiology, *N* (%)	12 (3)
Positive nasopharyngeal cultures, *N* (%)	4 (1)
Positive blood cultures, *N* (%)	0 (0)

*CRP* C-reactive protein, *IQR* interquartile range, *NIHSS* National Institutes of Health Stroke Scale, *SD* standard deviation

In the multiple linear regression analysis, NIHSS score, female gender, rectal (as opposed to tympanic) temperature, positive swallowing test (indicating dysphagia), intubation, CRP > 50/signs of infection at admission, and antipyretic treatment within 48 h were positively associated with peak body temperature within 48 h. These variables are presented in Table 3, together with the variables that remained in the last step of the regression analysis. Variables excluded from the preceding backward multiple regression analysis are presented in Table 4, in the order of exclusion. Rsquared (r^2^) was 0.355 for the entire multiple- regression model.

**Table 3:** Final results of the multiple linear regression analysis

	Regression coefficient	95% confidence interval	*P*-value	VIF
(Constant)	37.081	36.929–37.232	**0.000**	
NIHSS score	0.022	0.010–0.034	**0.000**	1.386
Antipyretic treatment within 48 h	0.563	0.432–0.694	**0.000**	1.120
Tympanic/Non rectal temperature reading	-0.265	−0.401- -0.129	**0.000**	1.087
CRP > 50 or signs of infection at admission	0.298	0.122–0.473	**0.001**	1.090
Unknown CRP level without signs of infection	0.047	−0.298-0.392	0.789	1.047
Positive swallowing test	0.335	0.130–0.540	**0.001**	1.054
Female gender	0.157	0.031–0.284	**0.014**	1.036
Intubation	0.470	0.044–0.896	**0.031**	1.379
Diabetes	0.142	−0.009-0.293	0.065	1.037
Smoking	−0.175	−0.371-0.020	0.079	1.037
Right sided stroke	−0.087	−0.222-0.048	0.204	1.162
Hemisphere not known	0.037	−0.163-0.238	0.714	1.171

*CRP* C-reactive protein, *NIHSS* National Institutes of Health Stroke Scale, *VIF* variance inflation factor

Significant *p*-values (<0.05) are written in boldface

**Table 4:** Variables excluded from the preceding backward multiple linear regression analysis, in order of exclusion

Variable	*P*-value at exclusion
Chronic infection	0.887
Hypertension	0.872
Unknown CRP level without signs of infection	0.848
Thrombolysis	0.822
Hyperlipidemia	0.783
Coronary heart disease/heart failure	0.800
Hemisphere not reported	0.709
Infarction	0.647
Chronic inflammatory disease	0.630
Previous stroke/TIA	0.355
Chronic obstructive pulmonary disease/ other chronic lung disease	0.267
Malignancy	0.264
Age	0.210
Atrial fibrillation	0.228

*CRP* C-reactive protein, *TIA* transient ischemic attack

## Discussion

In this retrospective study we confirm that body temperature elevation within 48 h of stroke onset is common and associated with neurological status at admission (NIHSS score), female gender, swallowing difficulties, intubation and CRP > 50 or signs of infection at admission.

As hypothesized, in the current study, symptom severity as determined by NIHSS score was a strong predictor of increased body temperature, which is also in concordance with several previous studies [1, 9–11]. This association could have several explanations that are not mutually exclusive. Firstly, it is possible that the initial elevation of body temperature is partly non-infectious; either due to local effects on cerebral thermoregulatory centers or to stimulation of the immune system caused by necrosis, as would be expected with a necrotic process in any organ [6, 12]. If local effects on thermoregulatory centers were responsible for the elevation in temperature, an association between stroke location and temperature elevation would be expected [6]. Unfortunately, such analyses could not be performed on the current data because of the inclusion of a large number of patients with ischemic stroke, in many of whom exact lesion location was unknown. A second possibility is that the stroke makes the patient prone to develop infections and that this effect is more pronounced with larger lesions. In a previous study, the NIHSS score was shown to predict post-stroke infections [13]. Stroke is known to make the patient susceptible to infections by several mechanisms, including immunosuppression [14], dysphagia [15] and urinary incontinence, prompting use of urinary catheters [16]. The strong relation between stroke severity and post-stroke body temperature elevation may explain in part the previously described association between temperature and stroke outcome, and emphasizes the importance of meticulous confounder control when investigating such relations.

The second hypothesis, that intracerebral hemorrhage would cause more pronounced temperature elevations than ischemic stroke, was not corroborated. We assumed that initial temperature elevation would mainly be due to local effects of the brain lesion, and that mechanisms for non-infectious temperature elevation caused by the lesion’s direct effects on thermoregulation in the brain would differ between ischemia and hemorrhage [5, 17, 18]. Furthermore, there is a widespread notion among stroke physicians that hemorrhagic stroke patients are more prone to fever. Surprisingly, in our study population, whether the patient had ischemia or hemorrhage was not associated to peak body temperature within 48 h of stroke onset. In other words, patients with hemorrhage did not have higher temperature than patients with ischemic stroke after controlling for the other factors. The factor was excluded in the preceding backward analysis with a *p*-value of 0.647, which in combination with the large study sample indicates that the risk for a type II error was very low. Previous studies investigating stroke severity and its correlation to post-stroke events either included ischemic or hemorrhagic stroke, or did not adequately control for stroke severity, and therefore this question has hitherto been left unanswered [1, 18]. The current lack of association between stroke subtype and temperature does not allow conclusions to be drawn regarding the pathophysiology of post-stroke body temperature .

Several other observations were made in the current study, which are, however, unrelated to the main hypothesis and therefore should be interpreted with caution. Firstly, an increased susceptibility to elevation in temperature in females was noted, in line with Saini et al. who observed that female gender was an independent predictor of elevated temperature within 24 h [1]. This could be explained by differences in temperature regulation between the sexes, since women generally seem to have a higher body temperature [19]. In the current regression analysis, a positive swallowing test and intubation were associated with peak body temperature within 48 h, which seems reasonable given the previously known connection between swallowing difficulties and respiratory infections after stroke onset [15, 20]. Furthermore, in the current study, age was surprisingly not associated with increased temperature. This is in contrast to Yan et al. who claimed that age > 65 predisposes for post-stroke fever [8]. Further, Ribeiro et al. concluded that NIHSS score and high age are correlated to post-stroke pneumonia [21]. A possible explanation for the current findings is that although elderly patients have a higher risk for post-stroke infections, their generally lower body temperature [22] might compensate for possible temperature elevations due to infections.

The study has some limitations that need to be emphasized. Several patients were excluded due to temperature readings more than 48 h from stroke onset or unknown timing of stroke onset, which might imply that the cohort is less representative for the study population. Another important limitation of this retrospective study regards determining patients’ peak temperature. According to the inclusion criteria, at least one temperature reading within 48 h of stroke onset was required for inclusion. However, because measurements were generally not performed more than three times a day, and in some patients only once daily, the recorded temperature readings may not reflect the patients’ temperature peaks. Another weakness of the study is that only in a few cases was neurologic status noted as NIHSS score directly by the examiner. The majority of the NIHSS scores were instead retrospectively calculated to NIHSS based on the description of neurologic status in the patient records, which is a source of error even if performed in accordance with a strict protocol. Previous disability was also retrospectively computed and subtracted from the total NIHSS to achieve NIHSS for the current stroke, which, consequently might be a weakness since it requires careful documentation in the medical records. In an effort to separate non-infectious temperature elevation from temperature elevation with infectious etiology, signs of infection during hospitalization were summarized in Table 2. The majority of patients with positive signs of infection and treatment with antibiotics had temperature elevation within 48 h. However, only a minority of patients with temperature elevations were subjected to lung radiology, blood cultures, or other investigations to pinpoint infections, making our estimate of the rate of infections extremely uncertain. Previous studies indicate that post-stroke infections occur between 1 and 7 days after stroke onset [9, 15, 23]; in the present study, few patients received antibiotics within the first 48 h from admission (median time to antibiotic treatment was 3 days). As well as weaknesses, the current study also has several strengths. First of all, the in-depth evaluation of the medical records of a relatively large patient cohort can be considered a strength. Further, the multiple regression analysis makes it possible to adjust for confounding factors. Although predicting factors for post-stroke temperature elevation have been investigated in several previous studies, few have focused on factors associated with body temperature within 48 h of both ischemic and hemorrhagic stroke.

## Conclusions

In accordance with the initial hypothesis, post-stroke temperature elevation seems to be associated with NIHSS status at admission as well as with gender, swallowing difficulties, intubation and CRP >50 or signs of infection at admission, but not with hemorrhage or ischemic stroke. The strong association with stroke severity may, at least partly, explain the association between post-stroke temperature elevation and less favorable outcome seen in previous studies [1–3].

## Abbreviations

CRP: C-reactive protein
ICD: International classification of diseases and related health problems
IQR: Interquartile range
NIHSS: National institute of health stroke scale
RC: Regression coefficient
SD: Standard deviation
TIA: Transient ischemic attack
VIF: Variance inflation factor
